# Enzyme Promiscuity in Serotonin Biosynthesis, From Bacteria to Plants and Humans

**DOI:** 10.3389/fmicb.2022.873555

**Published:** 2022-04-14

**Authors:** Sara Gonçalves, Daniela Nunes-Costa, Sandra Morais Cardoso, Nuno Empadinhas, John David Marugg

**Affiliations:** ^1^CNC—Center for Neuroscience and Cell Biology, CIBB—Center for Innovative Biomedicine and Biotechnology, University of Coimbra, Coimbra, Portugal; ^2^PhD Program in Experimental Biology and Biomedicine (PDBEB), Institute for Interdisciplinary Research, University of Coimbra, Coimbra, Portugal; ^3^Institute of Cell and Molecular Biology, Faculty of Medicine, University of Coimbra, Coimbra, Portugal; ^4^IIIUC—Institute for Interdisciplinary Research, University of Coimbra, Coimbra, Portugal

**Keywords:** serotonin, melatonin, biosynthetic pathways, substrate promiscuity, aromatic amino acid hydroxylase, aromatic amino acid decarboxylase

## Abstract

Serotonin is a phylogenetically ancient compound found in animals, plants, and some bacteria. In eukaryotes, serotonin is synthesized from the aromatic amino acid tryptophan *via* the key enzymes aromatic amino acid hydroxylase (AAAH) and aromatic amino acid decarboxylase (AAAD). Serotonin is also an intermediate in the melatonin biosynthetic pathway and is involved in several vital functions. In humans, serotonin is produced in the gut and in the brain, is critical in the regulation of multiple body functions, and its depletion has been implicated in multiple neurological disorders including depression and Alzheimer’s disease, as well as other peripheral conditions namely irritable bowel syndrome and fibromyalgia. The serotonin biosynthetic pathway is well described in eukaryotes, but very little is known about this pathway in bacteria. Evidence points to similar pathways since eukaryote-like AAAH and AAAD (and their genes) have been identified in multiple bacteria, even though serotonin production has not yet been detected in most species. Although data on bacterial tryptophan decarboxylase genes are very limited and no bacterial tryptophan hydroxylase genes have been identified to date, evidence suggests that serotonin production in bacteria might occur through different AAAH and AAAD. Substrate promiscuity in these enzymes has been previously reported and seems to be the key aspect in bacterial serotonin synthesis. Considering the human gut microbiota as a potential source of serotonin, further investigation on its biosynthetic pathways in microbes might lead to important discoveries, which may ultimately foster the development of new therapeutic strategies to treat serotonin depletion-related disorders in humans.

## Introduction

Serotonin, also known as 5-hydroxytryptamine (5-HT), is a multi-functional indolamine whose structure contains an indole ring that can capture light, inherited from its precursor L-tryptophan, which played a key role in converting solar energy into biochemical energy within primitive anaerobic unicellular organisms, through carboxylase action (Cox and Lee, 2016). Oxygen production, a consequence of anaerobic metabolism, resulted in the accumulation of reactive oxygen species (ROS) inside the cells, which caused carboxylases to acquire their hydroxylase function as a mechanism of protection against the reactive oxidizing agents. These primitive hydroxylases used tryptophan, tyrosine and phenylalanine as substrates, binding oxygen to them and forming different antioxidant compounds, such as serotonin. Thus, serotonin is a phylogenetically ancient compound, and its biosynthesis has been conserved throughout evolution in different phyla, from primitive forms of life, like unicellular organisms, sponges and hydras, to the most complex ones, like plants and vertebrates (Yabut et al., 2019). Serotonin is also an intermediate in the biosynthesis pathway for melatonin (*N*-acetyl-5-methoxytryptamine), a methoxyindole that was first isolated from the bovine pineal gland in 1958, and later identified in various other animal tissues and in almost all living organisms including plants, fungi and bacteria (Tan et al., 2016). Just like serotonin, melatonin is a very ancient molecule that was already present in primitive organisms, such as cyanobacteria and α-proteobacteria, in which it was a vital antioxidant and free radical scavenger (Zhao et al., 2019). The principle that serotonin and melatonin biosynthesis was conserved throughout evolution can be explained by the endosymbiotic theory in which cyanobacteria and α-proteobacteria were engulfed by primitive eukaryotes, eventually establishing a symbiotic association with the host, and later evolving into chloroplasts and mitochondria, respectively. Ultimately, divergent evolution caused the serotonin and melatonin functions, biosynthetic pathways, generation sites and regulation, to diverge between organisms (Zhao et al., 2019). Although serotonin was first identified about 70 years ago because of its constricting effects on smooth muscle (Rapport et al., 1948), today it is known to take part in most central and peripheral functions in the human body (Hensler, 2012). In the central nervous system, serotonin acts as a neurotransmitter involved in the regulation of multiple brain functions including sleep, mood, cognition, memory and sexual behavior. Peripherally, serotonin is associated with virtually all major organ systems and has a critical role in energy balance, appetite, gut motility, immunity, liver repair (Yabut et al., 2019) and cardiovascular and pulmonary physiology, among others (Berger et al., 2009). Melatonin physiologically works as an endogenous synchronizer that stabilizes circadian rhythms and therefore regulates, either directly or indirectly, multiple body functions and mechanisms that depend on that periodicity, such as body temperature, induction of sleep, blood pressure, immune responses, cell regulation and antioxidant protection (Claustrat and Leston, 2015). Furthermore, gut-produced melatonin and the metabolites that result from its degradation may exert diverse effects locally, particularly in the gut microbiota. Melatonin has important antimicrobial properties and can mediate activation and proliferation of intestinal mucosal immune cells, as well as modulation of microbial components and their effects on the human body rhythm system (Ma et al., 2019). Serotonin and melatonin functions in animals, plants and bacteria are summarized in Table 1.

**Table 1:** Serotonin and melatonin functions in animals, plants, and bacteria.

	Serotonin	Melatonin	References
Animals	CNS and ENS neurotransmission Metabolic, physiological, cardiovascular, gastrointestinal, and neurological regulation Immunomodulation	Crosstalk between CNS and ENS Regulation of circadian rhythm Modulation of gut mucosal immunity Antimicrobial action Modulation of the effects of gut microbiota activity signals on body rhythm system	Yabut et al., 2019 Ma et al., 2019
Plants	Growth and development regulation Reproduction and senescence Stress survival Antioxidant protection	Growth and development regulation Stress resistance Antioxidant protection Circadian rhythm regulation	Erland et al., 2019 Zhao et al., 2019 Pelagio-Flores and López-Bucio, 2016
Bacteria	Growth and development regulation Stress resistance Cell–cell communication Biofilm formation and quorum sensing	Regulation of circadian rhythm Plant-microbe interaction Antioxidant protection	Erland et al., 2019 Knecht et al., 2016 Danilovich et al., 2021

## Serotonin Biosynthesis in Animals

**Figure 1:**
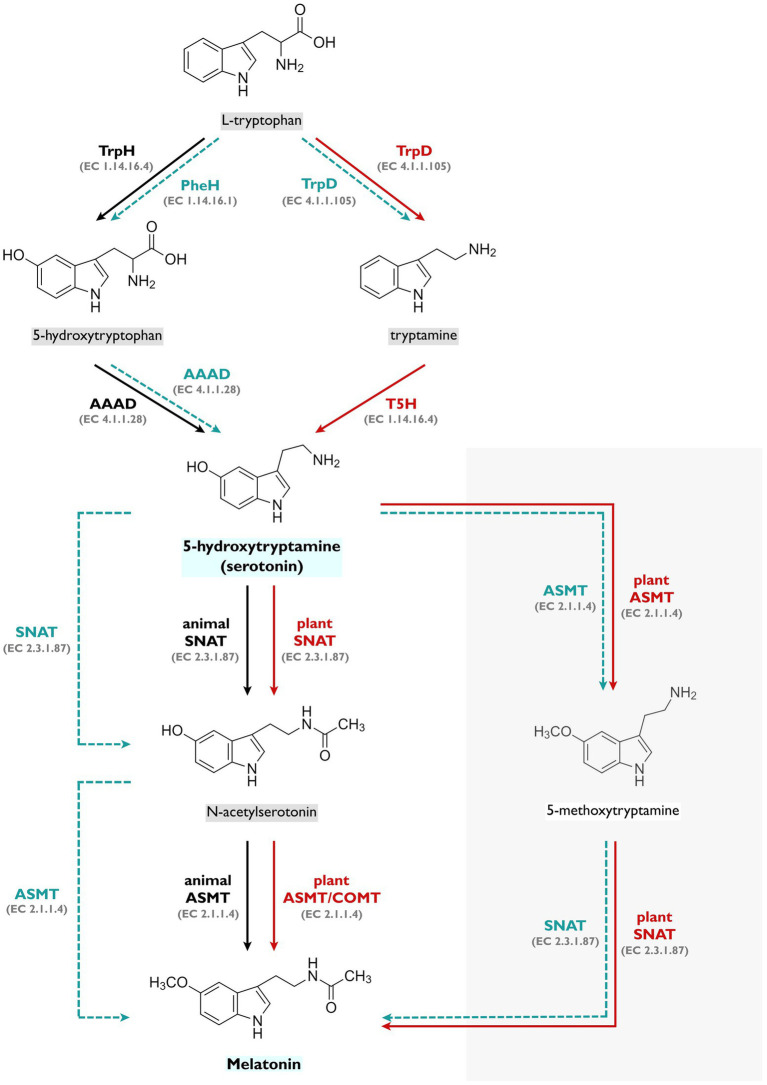
Serotonin and melatonin biosynthetic pathways. In animals (black arrows), L-tryptophan is hydroxylated by tryptophan hydroxylase (TrpH; EC 1.14.16.4) to form 5-hydroxytryptophan, which is then decarboxylated by aromatic amino acid decarboxylase (AAAD; EC 4.1.1.28) to produce serotonin. From serotonin, *N*-acetylserotonin is generated by serotonin-*N*-acetyltransferase (SNAT; EC 2.3.1.87), and finally into melatonin, by *N*-acetylserotonin *O*-methyltransferase (ASMT; EC 2.1.1.4; Yabut et al., 2019). In plants (red arrows), L-tryptophan is decarboxylated by tryptophan decarboxylase (TrpD; EC 4.1.1.105) to form tryptamine, which is then hydroxylated by tryptamine/tryptophan 5-hydroxylase (T5H, EC 1.14.16.4) to produce serotonin. Serotonin is then converted into *N*-acetylserotonin by serotonin-*N*-acetyltransferase (SNAT; EC 2.3.1.87), and finally into melatonin, by *N*-acetylserotonin *O*-methyltransferase (ASMT; EC 2.1.1.4) or by caffeic *O*-methyltransferase (COMT; EC 2.1.1.4). Alternatively, melatonin can also be synthetized *via* 5-methoxytryptamine in plants (Tan et al., 2016). Light blue dashed arrows represent putative serotonin and melatonin biosynthetic pathways for bacteria - tryptophan conversion into serotonin *via* 5-hydroxytryptophan, through phenylalanine hydroxylase (PheH; EC 1.14.16.1; Lin et al., 2014; Jiao et al., 2021) and bacterial AAAD activity (Koyanagi et al., 2012), and serotonin conversion into melatonin *via* 5-methoxytryptamine or *N*-acetylserotonin, through bacterial ASMT and SNAT enzymes (Tan et al., 2016; Ma et al., 2017; Jiao et al., 2021). Alternatively, tryptophan conversion into tryptamine by bacterial tryptophan decarboxylase (TrpD; EC 4.1.1.105) has also been detected before.

In humans and other animals, serotonin is synthesized from the amino acid L-tryptophan in a two-step reaction. L-tryptophan is initially hydroxylated by an aromatic amino acid hydroxylase (AAAH) to form 5-hydroxytryptophan (5-HTP), which is then decarboxylated by an aromatic amino acid decarboxylase (AAAD) to produce serotonin (5-HT; Yabut et al., 2019; Figure 1). Therefore, this biosynthetic pathway is carried out by two essential enzymes: AAAH and AAAD. AAAH are non-heme ferrous iron and tetrahydrobiopterin-dependent monooxygenases that use oxygen to hydroxylate their substrates. They include tryptophan hydroxylase (TrpH), phenylalanine hydroxylase (PheH) and tyrosine hydroxylase (TyrH). These three enzymes catalyze three general vital reactions that result in the conversion of tryptophan, phenylalanine, and tyrosine into 5-HTP, tyrosine and L-DOPA, respectively. AAAD are pyridoxal 5′-phosphate (PLP)-dependent enzymes that convert aromatic amino acids into amines (Koyanagi et al., 2012). AAAD catalyze multiple reactions including the decarboxylation of L-DOPA into dopamine by DOPA decarboxylase; 5-HTP into serotonin by 5-HTP decarboxylase; tryptophan into tryptamine by tryptophan decarboxylase (TrpD); phenylalanine into phenylethylamine (PEA) by phenylalanine decarboxylase; tyrosine into tyramine by tyrosine decarboxylase (TyrD); histidine into histamine by histidine decarboxylase, among others (Wishart et al., 2018). Serotonin is also an intermediate in the melatonin biosynthetic pathway, in which it is first acetylated into *N*-acetylserotonin by serotonin-*N*-acetyltransferase (SNAT), and then into melatonin, by *N*-acetylserotonin *O*-methyltransferase (ASMT; Pelagio-Flores and López-Bucio, 2016). The serotonin and melatonin biosynthetic reactions are illustrated in Figure 1. These biosynthetic pathways are limited by tryptophan availability and by the rate limiting enzymes TrpH and SNAT. Tryptophan hydroxylase activity requires tetrahydrobiopterin, oxygen, NADPH + H^+^ and a metal (Athar, 2010). SNAT’s mRNA expression in the pineal gland is under the influence of day/night cycles, causing this enzyme to only be active in specific periods of time and conditions (Claustrat and Leston, 2015).

Serotonin is produced in very low quantities in animals, mainly due to tryptophan limitation, as this amino acid can only be obtained through the diet and a large percentage is used in kynurenine synthesis (Yabut et al., 2019). Indeed, in the kynurenine pathway, tryptophan is oxidized by indoleamine 2,3-dioxygenase 1 (IDO-1), an enzyme mainly found in macrophages, microglia, neurons and astrocytes that is up-regulated by certain cytokines and inflammatory molecules, such as lipopolysaccharides, amyloid peptides and interferon gamma (IFN-γ), which justifies the observed depressive states in individuals whose diseases are associated with chronic inflammation, like cancer and neurological disorders (Yabut et al., 2019). Over 90% of total human body serotonin is generated and located in the gut (Martin et al., 2019), with the remaining 10% being produced in the brain and other peripheral sites, such as pancreatic β-cells, osteoclasts, and adipocytes. Central serotonin and peripheral serotonin are considered two separate pools because these molecules are synthesized by two distinct TrpH isoforms (TrpH1 in non-neuronal cells and TrpH2 in neuronal cells) and 5-HT cannot cross the blood–brain barrier (Spohn and Mawe, 2017). In the gut, enterochromaffin cells (EC) synthesize serotonin using TrpH1, whose activity and expression appears to be regulated by nearby cells and certain nutrients, such as carbohydrates (Yabut et al., 2019). Serotonin is then released in a mechanical and chemical stimulus-regulated way (Banskota et al., 2019) and takes part in multiple reactions, not only in the intestine, but also systemically by binding to circulating platelets in the bloodstream (Yabut et al., 2019). In the CNS, serotonergic neurons produce serotonin *via* TrpH2 activity, an isoform also used by enteric neurons in the enteric nervous system (ENS; Bader, 2019). Serotonin synthesis in the brain is highly limited by the quantity of tryptophan that crosses the brain–blood barrier as only free/unbound plasma tryptophan can do so (Athar, 2010). After synthesis, serotonin is encapsulated in vesicles and released by exocytosis into the neuronal synaptic cleft, activates specific receptors and is then removed from the synapse by presynaptic neurons reuptake (Gresch, 2013). This way, serotonin can be sent from the Raphe nuclei neurons in the brainstem, where serotonergic neurons originate, to nearly all areas of the CNS (Bader, 2019).

Melatonin secretion in humans is regulated by light/dark cycles and this photic information is transmitted through the retinohypothalamic tract to the suprachiasmatic nuclei of the hypothalamus, which in turn communicates with multiple areas in the brain, including the pineal gland, where melatonin is synthetized, but not exclusively. Studies estimate that about 99% of total body melatonin is produced in other tissues, although it is never released into the bloodstream and therefore its circulating quantities are not systemically significant. In the presence of light, during the day, melatonin synthesis in the pineal gland is inhibited and its levels peak at night, when it is released into circulation, reaching all body tissues, including the brain. Melatonin can cross the blood–brain barrier (BBB), and easily cross cell membranes because it is soluble in lipid and water (Claustrat and Leston, 2015). Melatonin receptors are widely distributed throughout the human body and, even though some effects of melatonin cannot be explained by membrane receptors or by ROS scavenging, many of its activities known to date are mediated by transmembrane G-protein coupled receptors, which activate signaling cascades in the target cells (Zhao et al., 2019).

## Serotonin Deficiency Disorders

As previously mentioned, the serotonergic system is involved in multiple CNS functions and its homeostasis is crucial to maintain proper brain function (Athar, 2010). Serotonergic neurons are also responsible for controlling ENS development, influencing neurogenesis and ensuring the survival of the new developing neurons. Although serotonin has a critical role in the CNS and ENS function, the highest percentage of serotonin is in the gut enterochromaffin cells (O’Mahony et al., 2015). Dysregulation of serotonergic signaling pathways has been previously implicated in many psychiatric and neurological conditions such as pain, sleep problems, appetite and mood variations (Athar, 2010), and disorders like depression, anxiety, schizophrenia, attention deficit hyperactivity disorder (ADHD), obsessive compulsive disorder (OCD), migraine headache, autism spectrum disorders (ASD; Pourhamzeh et al., 2021), bipolar disorder (BP) and addictive behavior (Lin et al., 2014). Serotonin depletion has also been implicated in neurodegenerative diseases, such as Alzheimer’s disease, as serotonin levels decrease with age and neuroinflammation processes have been associated with reduction of serotonin precursors in the CNS (Danilovich et al., 2021). Additionally, lower levels of serotonin have been detected in fibromyalgia patients, where this imbalance has also been associated with other key symptoms such as fatigue and cognitive deficits (Welsch et al., 2018). Outside the CNS, dysregulation of serotonin levels has been connected to gastrointestinal disorders. Studies report deviations in serotonin levels in inflammatory bowel disease (IBD) and colitis. Increased serotonin-producing EC quantity has been observed in Crohn’s disease (CD) and in ulcerative colitis (UC) patients. These gut inflammatory conditions have been linked to serotonin-dependent angiogenesis, as serotonin has angiokine function in endothelial cells and increased vascularity has been observed in colitis and IBD. Disruption of serotonin balance levels has also been previously associated with irritable bowel syndrome (IBS), celiac disease, neuroendocrine tumors, and metabolic disorders, such as obesity and type 2 diabetes (Banskota et al., 2019). Melatonin levels dysfunction is most associated with sleep disorders. However, as it controls multiple systemic circadian rhythm-dependent processes, its deregulation is also implicated in other conditions such as epilepsy, autism, anxiety, bipolar disorder, and depression (Danilovich et al., 2021).

Due to their vital functions on the human body, serotonin and melatonin pathways are therapeutically relevant in many disorders. Serotonergic system components (such as serotonin receptors) are commonly used as pharmacological targets, which has proven to be very effective in the treatment of some neuropsychiatric conditions such as depression, through selective serotonin reuptake inhibitor drugs. Melatonin is extensively used to treat sleep-associated problems, such as insomnia and jetlag, through oral administration. Pioneering studies also evidenced its beneficial effects on prevention and treatment of age-associated neurodegenerative diseases, like Alzheimer’s, and Parkinson’s diseases, due to its anti-inflammatory and antioxidant properties. Clinical studies with cancer patients revealed that oral administration of melatonin decreases the toxicity of chemotherapeutic drugs. Due to its cellular protection and damage repair activity, melatonin has also shown to be effective in preventing tumorigenic mutations in animal models of cancer. Moreover, this indolamine has also been indicated as an effective coadjuvant therapeutic in gut diseases and in parasitic infections, due to its link with the immune system (Danilovich et al., 2021).

## The Microbiome-gut-brain Axis and the Serotoninergic System

The gut microbiome comprehends the complex and dynamic microbial community present in the mammalian gastrointestinal tract (Galland, 2014) where it functions as a “metaorganism” with a symbiotic and commensal relationship with the mammalian host (Hill et al., 2014). Human gut microbial cells are estimated to be at a ratio of approximately 1:1 with our own cells and over 9 million genes have been identified so far. The human gut microbial community, whose composition varies between individuals and is influenced by intrinsic and external factors (Thursby and Juge, 2017), holds up to thousands of different species from which about 90%–99% are anaerobic bacteria, with the remaining population composed of archaea, viruses, fungi and protozoa (Hill et al., 2014). The gut microbiome has been gathering more and more attention by the scientific community in the last decade as numerous studies continue to uncover its vast impact on human health through mechanisms not yet fully elucidated. Its protecting, structural and metabolic functions, such as food processing, pathogen displacement and synthesis of vitamins, are not restricted to the gut and the ENS, as the microbial-produced and regulated compounds are released into the bloodstream, reaching and acting at other distal organs and tissues, exerting important endocrine effects as well (Clarke et al., 2014). Recently, the gut microbiome has been implicated as a critical element on the human gut–brain axis, which comprehends the bidirectional communication network between the ENS and the CNS, as many bacteria are capable of not only recognizing but also often synthesize many of the same neurochemicals as those produced by the host’s nervous system, thus possibly influencing the host CNS through immunologic, biochemical, neural or neuroendocrine mechanisms (Lyte, 2013). Overall, although the molecular cascades involved in these events are still being investigated, there may be three major ways by which the gut microbiome communicates with the human brain. The first route involves signaling *via* the vagus nerve, which is thought to be stimulated by microbial metabolites, namely neurotransmitters such as serotonin, fatty acids, and myriad molecules, and cause activity alterations in specific brain regions consequently affecting body processes. The second pathway involves microbial stimulation of immune cells to release cytokines into the bloodstream, ultimately influencing neurological processes in the CNS, such as microglia activation. The third mechanism is a combination of the previous two in which gut microbiota metabolites may travel to the brain *via* bloodstream or stimulate gut cells to produce compounds that will activate the terminal branches of the vagus nerve (Kanwal, 2016).

The noninfectious ability of these microbes to influence the host behavior has been reported in multiple studies and specific gut bacteria have been shown to cause specific behavioral changes. One of the first studies in that field consisted of oral administration of *Campylobacter jejuni* in mice, which resulted in anxiety-like behavior, through vagus nerve communication (Lyte, 2013). Indeed, germ-free mice show development and physiological irregularities, which are reversible by early life colonization with gut bacteria. Stress responses also seem to be affected by the gut microbiome as studies with germ-free mice showed that these rodents were less timid and vigilant comparatively to conventional mice, possibly affecting their survival instincts (Galland, 2014). In turn, the gut microbiota itself is also affected by the host and can interact with host-derived compounds. This duality of influencing and being influenced by the host, leads the microbiota to take part in the regulation of complex endocrine networks, virtually functioning as an “endocrine organ” (Clarke et al., 2014). Impairment of the microbiome–gut–brain interaction has been associated with multiple CNS and ENS related disorders such as ASD, anxiety, depression, Parkinson’s disease, and IBS (Israelyan and Margolis, 2018).

Recent studies have revealed the direct and indirect effects of gut bacteria activity on tryptophan metabolism and the serotonergic system (O’Mahony et al., 2015) and although it has been determined that gut microbes play a very important role in their regulation, the exact mechanisms through which that occurs are still to be elucidated (Yano et al., 2015). Those effects have been strongly hypothesized to occur through bacterial manipulation of the host serotonergic system pathways and its intervening molecules’ levels, particularly tryptophan (Knecht et al., 2016). It has been previously demonstrated that germ-free mice have low blood and colon levels of serotonin. Interestingly, it was discovered that serotonin levels in the mice intestinal epithelium and lumen are regulated by *Turicibacter sanguinis*, which uses a membrane transporter to import serotonin, through a mechanism that favors its colonization (Fung et al., 2019). The bacterium can alter the host’s expression of metabolic pathways involved in lipid and steroid metabolism, which is accompanied by a reduction in systemic triglyceride levels and inguinal adipocyte size. This example highlights the ability of some gut bacteria to communicate bidirectionally with the host’s serotonergic system to promote their own colonization. Some microbial secondary metabolites might also impact serotonin levels, such as short-chain fatty acids (SCFA), which influence Tph1 mRNA levels in EC cells, resulting in higher intestinal serotonin levels. Fecal metabolites produced by Clostridial species also seem to increase serotonin levels in EC cell cultures and in germ-free mice (Israelyan and Margolis, 2018). The bacterial use of tryptophan in alternative dominant metabolic processes, such as the kynurenine pathway, may also be responsible for decreasing tryptophan availability in the host’s gut (Yano et al., 2015). This is supported by studies with germ-free mice where elevated plasma tryptophan levels could be normalized after gut microbial colonization (Clarke et al., 2014).

## Serotonin and Melatonin in Plants and OTHER EUKARYOTIC ORGANISMS

Serotonin (phytoserotonin) production in plants was first described in 1954 in *Mucuna pruriens* (Bowden et al., 1954). Melatonin (phytomelatonin) in plants was only reported later in 1993 in *Pharbitis nil* and *Solanum lycopersium* (Vantassel et al., 1993). Since then, these compounds have been identified in multiple other plant species where their levels are highly variable, depending on the species and on the plant tissue (Kang et al., 2007). Phytoserotonin appears to be highly important throughout all plant life stages, including germination, growth, reproduction, and senescence, as well as stress survival and tolerance (Erland et al., 2019). Its average content is estimated to be 100× higher than that in the animal brain (Azmitia, 2007) and its levels can go up to 400 μg/g in walnuts (*Juglans regia*; Pelagio-Flores and López-Bucio, 2016). Phytomelatonin is also intimately involved in the regulation of multiple plant life processes such as seed protection, germination, vegetative growth, root development, fruit maturation and senescence delay (Zhao et al., 2019) and has previously been associated with circadian rhythm regulation (Pelagio-Flores and López-Bucio, 2016). Alike phytoserotonin, phytomelatonin plays a very important role in stress tolerance as well (Zhao et al., 2019). Melatonin levels have been reported to go up to 230 μg/g in Pistachio kernels (Pelagio-Flores and López-Bucio, 2016). Phytoserotonin and phytomelatonin functions in plants are summarized in Table 1.

Contrary to animals, plants retained the ability to synthetize tryptophan during evolution (Zhao et al., 2019). Additionally, plants possess mitochondria as well as chloroplasts, both of which have been confirmed to be important melatonin biosynthesis sites (Wang et al., 2017). These aspects combined seem to make the biosynthetic processes of this indolamine more productive, when compared to animals. Evolution wise, these features were probably crucial since plants, as immobile beings, cannot behaviorally escape environmental threats. For that reason, they need to respond more quickly and more strongly to those stressful conditions to survive. Those stressful conditions boost ROS production and oxidative damage, triggering defense mechanisms such as antioxidant production. Multiple studies have shown that melatonin levels rise in response to various environmental insults (Zhao et al., 2019) and that serotonin levels are higher in plant tissues undergoing stress (Erland et al., 2019).

The serotonin and melatonin synthetic pathways in plants differ from those in humans and other vertebrates. While in vertebrates, serotonin biosynthesis occurs through L-tryptophan hydroxylation followed by decarboxylation, in plants, the order of the steps in the process is inversed and L-tryptophan is first decarboxylated into tryptamine by tryptophan decarboxylase, followed by hydroxylation of tryptamine into serotonin by tryptamine 5-hydroxylase (T5H; Figure 1; Kang et al., 2007). Serotonin may then be converted into *N*-acetylserotonin, by serotonin-*N*-acetyltransferase (SNAT), and then into melatonin by *N*-acetylserotonin *O*-methyltransferase (ASMT). Although the conversion of serotonin into melatonin is similar in animals and plants, SNAT and ASMT are not homologous between these groups (Erland et al., 2019).

However, the biosynthetic pathway described before may not be universal to all plants as some studies reported possible alternative pathways. Serotonin synthesis *via* 5-HTP, instead of tryptamine, was reported in *Hypericum perforatum* (Murch et al., 2000). Also in plants, multiple pathways have been suggested for melatonin biosynthesis, in which different enzymes may be involved and in an apparent species-dependent way (Back et al., 2016). Tan et al. (2016) proposed melatonin synthesis to occur *via* 5-methoxytryptamine (5-MT), in which serotonin would first be *O*-methylated to 5-MT and the latter then *N*-acetylated to melatonin, as shown in Figure 1. *Arabidopsis thaliana* SNAT has shown higher affinity for 5-MT than for serotonin. Even though this has been hypothesized to be the predominant melatonin synthetic pathway in plants, it should not be generalized to all plants as multiple isoforms of ASMT and SNAT have been detected in different species and, in some cases, other enzymes such as caffeic acid *O*-methyltransferase (COMT) may be involved in the pathway as well. This pathway has also been detected in yeasts and cyanobacteria, in which SNAT also demonstrated higher catalytic efficiency with 5-MT than with serotonin. Since chloroplasts are the main localization of plant SNAT, and have been hypothesized to be originated from cyanobacteria, the melatonin synthetic pathway in plants is thought to be partially inherited from this group of bacteria (Tan et al., 2016). Consistently, melatonin production *via* 5-MT was also detected in *P. fluorescens*, although further research on bacterial ASMT and SNAT genes is needed to fully understand how these reactions really occur (Jiao et al., 2021).

Although it is known that serotonin and melatonin are used by virtually all organisms, there is little evidence on their production in organisms other than animals and plants, as the greater part of the research is focused on these two groups. Melatonin has been previously detected in wine and beer, which is thought to arise from yeast metabolism namely during ethanol fermentation. Serotonin is also found in wines and its biosynthesis has been reported in yeasts exposed to UV radiation (Mas et al., 2014). The protozoan parasite *Entamoeba histolytica* secretes serotonin, which seems to be responsible for some of its infection symptoms in animals (McGowan et al., 1983). Serotonin biosynthesis in bacteria has been reported occasionally, although the mechanisms through which it occurs are not fully understood, as discussed in the next section.

## Biosynthesis of Serotonin in Bacteria

Although the physiological roles of serotonin and melatonin in bacteria and other microbes are not fully understood, they appear to be mostly related to defense mechanisms. Serotonin has been implicated in intercellular communication between microorganisms, growth regulation and protection against UV radiation. Exogenous serotonin seems to be important in biofilm formation in *Pseudomonas aeruginosa* through quorum sensing mechanisms (Knecht et al., 2016). Melatonin’s main role in microbes seems to be related to antioxidant protection, and it also seems to contribute to circadian rhythm regulation in certain human gut bacterial species, as dark conditions seem to lead to increasing levels of this molecule. As these indolamines have demonstrated such beneficial effects in microbial survival, it would be evolutionarily favorable for these organisms to retain the capacity to synthetize such important compounds (Danilovich et al., 2021). Serotonin and melatonin functions in bacteria are summarized in Table 1. However, scientific consensus about bacterial serotonin production is still absent even though AAAH and AAAD have been isolated from some bacteria and the presence of the corresponding genes has been confirmed in their genomes.

*De novo* serotonin production has been reported in some studies on bacterial biogenic amine (BA) formation using combinations of lactic acid bacteria and food-borne pathogens (Özoğul, 2004; Özogul et al., 2012). However, since part of these studies were performed in mixed cultures and were based on serotonin detection (and/or its biosynthetic intermediates) often in very low quantities in the culture medium, the overall evidence on the origin of these compounds is not very conclusive. Despite the various efforts that have been made for decades now, there is still no solid evidence of bacterial serotonin biosynthesis, as the data available on this topic are not very extensive and some of the available literature is ambiguous or vague (Lyte, 2013). Additionally, the simultaneous presence of AAAH and AAAD genes in the same bacterial strain is very rare and *Pseudomonas putida* KT2440 is one of such rare cases, in which both AAAH (Herrera and Ramos, 2007) and AAAD (Koyanagi et al., 2012) genes have been identified.

To date, we could only find very limited reports addressing the mechanisms of serotonin biosynthesis in bacteria. In an initial study, Ma et al. (2017) used isotope-labeled L-tryptophan to characterize the melatonin biosynthetic pathway in *Pseudomonas fluorescens* RG11, which resulted in detection of isotope-labeled serotonin and melatonin by HPLC-MS. Isotope-labeled 5-HTP, but not tryptamine, was also detected, suggesting that this specific strain possibly produces serotonin through an identical pathway to the one observed in vertebrates. However, the bacterial genes involved in this pathway were not investigated in the study and tryptophan hydroxylase genes were not identified in the published genome sequences of *P. fluorescens*, possibly indicating that either the gene is contained in a plasmid or that tryptophan hydroxylation is catalyzed by a different enzyme, such as phenylalanine hydroxylase, as suggested by the authors (Ma et al., 2017). Indeed, this enzyme has been previously confirmed to hydroxylate both phenylalanine and tryptophan in *P. fluorescens* (Lin et al., 2014) and the corresponding gene has been detected in multiple bacteria species (contrarily to tryptophan hydroxylase genes, which have not been identified in bacteria so far). Recently, in a follow-up study, the phenylalanine hydroxylase encoding gene from *P. fluorescens* RG11 was cloned and expressed (Jiao et al., 2021), and the corresponding enzyme also exhibited hydrolase activity with tryptophan as substrate, to form the serotonin precursor 5-HTP. Additionally, the team tested serotonin and melatonin production in a mutant strain lacking functional PheH, in which serotonin and 5-HTP production decreased considerably. These studies provide evidence that PheH may contribute to the first step of serotonin and melatonin synthesis, as represented in Figure 1, at least in this specific bacterial strain. For the conversion of 5-HTP to serotonin, a tryptophan decarboxylase (or AAAD) is required, but so far this enzyme has not been identified in *P. fluorescens.* Nonetheless, 5-HTP decarboxylation was observed in *P. putida* AAAD (Koyanagi et al., 2012), which could potentially be responsible for further conversion of 5-HTP into serotonin in these *Pseudomonas* species, but so far, this next step has not been confirmed yet.

Regarding bacterial AAAD, a great part of the currently existing research was performed in the scope of biogenic amines (which include serotonin, tyramine, tryptamine, and melatonin) production in fermented foods, mostly in lactic acid bacteria, due to their adverse effects on human health. It is known that at least some of the bacterial decarboxylase genes involved in BA synthesis are located in genomic islands or in unstable plasmids. Additionally, these genetic elements appear to be strain-specific rather than species-specific. Such observations led scientists to believe that the BA-producing ability of bacteria is a result of horizontal gene transfer that occurred between microbial organisms during evolution (Mohedano et al., 2015).

Tyramine, the product of tyrosine decarboxylation by tyrosine decarboxylase, is one of the most studied BA in this context due to its dangerous effects on the vascular system when ingested (Mohedano et al., 2015), and tyrosine decarboxylase genes have already been identified in multiple bacterial strains, such as *Enterococcus faecalis* (Pessione et al., 2009), *Enterococcus faecium*, *Enterococcus durans* (Burdychova and Komprda, 2007), *Lactococcus lactis*, *Lactobacillus brevis* (Fernandez et al., 2004) and *Carnobacterium divergens* (Coton et al., 2004). On the other hand, tyramine production has been reported in other bacteria, such as *Micrococcus* sp. (Nakazawa et al., 1977), *Leuconostoc* sp. and *Proteus mirabilis* (González de Llano et al., 1998), for which further genetic analyses on tyrosine decarboxylase genes are lacking to date.

Tryptamine, the product of tryptophan decarboxylation in plants, has been detected in cultures of some bacterial species such as *Lactococcus lactis*, *Leuconostoc* spp., *Proteus mirabilis* (González de Llano et al., 1998), *Micrococcus percitreus* (Nakazawa et al., 1977), *Bacillus cereus* (Perley and Stowe, 1966), *Hafnia alvei*, *Morganella morganii* and *Klebsiella pneumoniae* (Özoğul, 2004). However, in some of these studies it is not completely clear if this compound is indeed a product of bacterial metabolism. Williams and colleagues discovered and characterized two tryptophan decarboxylases from *Clostridium sporogenes* and *Ruminococcus gnavus* (Williams et al., 2014). Even though the *C. sporogenes* AAAD was also active towards tyrosine and the *R. gnavus* AAAD was capable of decarboxylating tyrosine and phenylalanine, both enzymes showed considerably higher catalytic efficiencies with tryptophan, hence their characterization as TrpD. Analysis of multiple human gut metagenomes revealed the existence of *R. gnavus* TrpD homologs in 9–17% of the examined samples. Tryptamine production by these Firmicutes may be particularly relevant to the host’s tryptophan metabolism as these bacteria may sequester tryptophan from the diet and limit its availability to the host (Williams et al., 2014). Overall, tryptamine formation in these bacterial species could suggest the existence of a plant-alike serotonin biosynthetic pathway in bacteria (represented in Figure 1), possibly in a strain-specific way. Further research and additional evidence are needed to corroborate this hypothesis.

So far, a few AAAH have been isolated from members of multiple bacterial genera, such as *Pseudomonas* and *Chromobacterium* (Lin et al., 2014). Microbial AAAH differ from the eukaryotic ones as they are usually monomeric, while mammalian hydroxylases are tetrameric. Microbial enzymes also lack an *N*-terminal extension of about 200 amino acids, as well as the C-terminal domain, which is involved in tetramerization in the eukaryotic enzymes (Zhao et al., 1994). Most of the AAAH bacterial genes discovered to date are annotated as phenylalanine hydroxylase genes (Jiao et al., 2021). AAAH genes from *Pseudomonas aeruginosa* (Zhao et al., 1994), *Chromobacterium violaceum* (Chen and Frey, 1998), *Colwellia psychrerythraea* (Leiros et al., 2007) and multiple *Chlamydia* species (Abromaitis et al., 2009) had homology with mammalian hydroxylases. Some of these genes, specifically PheH genes, seem to be contained in operons along with other enzymes such as dehydratases and aminotransferases in *P. aeruginosa* (Zhao et al., 1994), as well as transporters and regulatory proteins in *P. putida* (Herrera and Ramos, 2007).

Considering the current evidence on bacterial AAAH and AAAD, although tryptophan decarboxylation has been detected in several bacteria, the existent data on bacterial tryptophan-specific decarboxylase genes are very limited (Williams et al., 2014) and, to our knowledge, no bacterial tryptophan hydroxylase genes have been described so far. However, and despite their annotation, multiple reports have been demonstrating the capacity of these enzymes to use more than one substrate, including tryptophan, as discussed in the next section. The putative substrate promiscuity detected in some of these bacterial enzymes may be particularly relevant in some pathways, such as serotonin biosynthesis, which could possibly explain the lack of data on bacterial tryptophan hydroxylase and tryptophan decarboxylases.

## Bacterial AAAD and AAAH and Substrate Promiscuity

Enzyme substrate promiscuity is not a new concept. The widely spread capacity of enzymes to use diverse substrates and catalyze reactions leading to different products other than those they are specialized at producing under a given condition, has been known for a long time. It has been theorized that primordial enzymes might have had very broad specificities, acting on multiple substrates, thus granting them an expanded metabolic range. Evolutionarily, enzymes became more and more specialized, which improved their metabolic efficiency. On the other hand, the promiscuity found in present enzymes belonging to the same family might suggest their divergent evolution from a common ancestor (Khersonsky and Tawfik, 2010). Prokaryotic phenylalanine hydroxylase genes in particular, have been hypothesized to be the ancestors of hydroxylases that later diverged and originated animal TrpH, PheH and TyrH (Lin et al., 2014).

Eukaryotic AAAH and AAAD substrate promiscuity has been reported before. Because the biopterin-dependent AAAH share homologous catalytic cores, require the same cofactors (iron and pterin) and their aromatic substrates have relatively similar structures, it has been theorized that the three hydroxylases will use all three substrates, at least to some extent (Fitzpatrick, 1999). For instance, human TrpH and PheH have been shown to hydroxylate both tryptophan and phenylalanine with similar kinetics (McKinney et al., 2001). As for AAAD, and although they all seem to be evolutionarily related, plant AAAD usually exhibit increased specificity, in contrast to animal AAAD, that typically seem to be able to use different substrates (Kawalleck et al., 1993).

During the last decades, some studies reporting the possible misannotation of some bacterial AAAD have been emerging. Koyanagi et al. (2012) reported high L-DOPA specificity for a *Pseudomonas putida* AAAD, whose encoding gene was previously annotated as tyrosine decarboxylase gene. This same “highly-specific” L-DOPA decarboxylase also showed activity towards 5-HTP, though with lower catalytic efficiency (Koyanagi et al., 2012). The capacity of this L-DOPA decarboxylase to use 5-HTP as substrate may be relevant in the putative 5-HTP decarboxylation step in the serotonin biosynthetic pathway (Figure 1), even if the catalytic efficiency is much lower for this substrate. A tyrosine decarboxylase gene from *Lactobacillus brevis* was cloned and expressed, and demonstrated enzymatic activity with tyrosine and L-DOPA, but not glutamate, despite sharing 100% amino acid sequence identity with an enzyme previously annotated as glutamate decarboxylase from a different *L. brevis* strain (Zhang and Ni, 2014). Multiple tyramine-producing Gram-positive bacteria, such as *E. faecalis*, *E. faecium* and *C. divergens*, share a genetic cluster organization in which the tyrosine decarboxylase gene (*TyrD*) is present, along with tyrosine/tyramine antiporter (*TyrP*), tyrosyl-tRNA synthetase (*TyrS*) and Na+/H+ antiporter (*nhaC-2*) genes. Tyrosine decarboxylase from *E. faecium* has been experimentally shown to be responsible for the decarboxylation of both tyrosine and phenylalanine into tyramine and PEA, respectively. The simultaneous tyramine and PEA production seems to be shared among other gram-positive bacterial strains where the tyrosine decarboxylase gene is present as well, namely other *Enterococcus* and *Lactobacillus* species (Marcobal et al., 2012). More recently, a study focused on Parkinson’s disease, uncovered the pivotal role of the tyrosine decarboxylase gene in the conversion of L-DOPA into dopamine in several human gut *Enterococcus* and *Lactobacillus* strains (van Kessel et al., 2019), which was a crucial finding to explain the increased dosage regimen of L-DOPA treatment required in some Parkinson’s disease patients.

Although tyrosine decarboxylase activity has been extensively studied in bacteria, not much is known about tryptophan decarboxylation. Tryptophan decarboxylase activity has been reported in *Micrococcus percitreus*, along with tryptamine formation (Nakazawa et al., 1977). This AAAD also demonstrated activity towards 5-HTP, L-DOPA, as well as tyrosine and phenylalanine, which inhibited tryptophan decarboxylation through uncompetitive inhibition mechanisms (Nakazawa et al., 1987). However, there is no record of genetic characterization for this AAAD. As previously mentioned, the analyzed *C. sporogenes* AAAD was more efficient at decarboxylating tryptophan and was therefore characterized as tryptophan decarboxylase, even though the enzyme’s gene sequence had been previously annotated as a tyrosine decarboxylase gene (Williams et al., 2014). So far, this is the only study claiming identification of a tryptophan decarboxylase in bacteria. However, the assay used in the study is only qualitative and some experimental issues regarding limited solubility of tyrosine are mentioned, which might have compromised the accuracy of the results. Overall, the substrate promiscuity observed in bacterial tyrosine decarboxylases leads to the possibility that this enzyme might also be able to use tryptophan as a substrate and potentially contribute to serotonin synthesis.

Tryptophan hydroxylation by PheH has been reported in previous studies. However, the affinity of these enzymes for tryptophan is generally lower than that for phenylalanine. Protein engineering studies have shown that AAAH substrate preference can be shifted through substitution of just a small number of residues in the enzyme amino acid sequence, specifically residues that interact with enzyme cofactors and substrates (Jiao et al., 2021). Putative PheH from *Pseudomonas aeruginosa*, *Pseudomonas putida*, *Pseudomonas fluorescens*, *Ralstonia eutropha* and *Xanthomonas campestris* have shown small activity with tryptophan, although their substrate preference was stronger for phenylalanine. However, PheH catalytic efficiency with tryptophan could be enhanced through site-directed mutagenesis (through substitution of specific residues identified in PheH with the respective residues conserved in TrpH) in PheH from *X. campestris*. In the same study, a highly active PheH was successfully engineered to convert tryptophan into 5-HTP in *E. coli*, using tetrahydromonapterin (MH_4_) and an improved recycling system for this cofactor, which is an endogenous *E. coli* metabolic product (Lin et al., 2014). In other studies, similar site-directed mutagenesis procedures were also successfully performed in PheH from *C. violaceum* (Kino et al., 2009) and PheH from *Cupriavidus taiwanensis* (Mora-Villalobos and Zeng, 2017), resulting in enhanced enzyme affinity for tryptophan and, consequently, higher tryptophan hydroxylase activity in *E. coli*.

Considering the available evidence on bacterial AAAH and AAAD substrate promiscuity, the fact that some of these enzymes can use tryptophan as substrate, even if the preference for other substrates is higher, should not be disregarded. In bacteria, this aspect may be particularly important (if not crucial) in serotonin biosynthesis as evidence seems to suggest that these reactions are catalyzed by multi-specific enzymes, different from those described for the classic serotonin biosynthesis pathways in animals and plants. From an evolutionary point of view, bacterial enzymes are more primitive and, as such, their specificities are likely broader when compared to eukaryotic ones, which are theorized to have gone through specialization processes over time (Khersonsky and Tawfik, 2010). Additionally, in natural biological conditions, the apparently low affinity of these enzymes for tryptophan may be physiologically sufficient for the processes in which they take part since in these bacteria (as well as in plants), tryptophan availability is much higher than for animals, due to their capability of synthetizing this amino acid (Zhao et al., 2019). Interestingly, substrate promiscuity stretches out beyond the serotonin synthesis pathway, as SNAT enzymes from cyanobacteria (Tan et al., 2016) have shown affinity for both serotonin and 5-MT.

## Concluding Remarks

Taking all the information discussed, evidence points to bacterial synthetic pathways for serotonin similar to the ones described for eukaryotes, as eukaryote-like AAAH and AAAD (as well as their genes) have been identified in multiple bacteria strains.

Considering the limited genetic evidence on bacterial tryptophan decarboxylases (Williams et al., 2014) and tryptophan hydroxylases, as well as the substrate promiscuity reported in bacterial AAAH and AAAD, bacterial biosynthesis of serotonin through other enzymes, such as PheH (as shown in Figure 1), becomes more plausible. Overall, evidence suggests an animal-like route (*via* 5-HTP) for serotonin synthesis in bacteria, but the plant-like route (through tryptamine) should not be discarded as these biosynthetic pathways seem to be very complex and might be species/strain-specific (Jiao et al., 2021). All in all, it is essential to take substrate promiscuity into account in future studies on bacterial serotonin and melatonin biosynthesis, as it seems to be the key aspect in all the given examples and may be extremely important to fill some of the knowledge gaps in current research.

There is still a long way to go in this field of research. The bacterial serotonin biosynthetic pathways are still very much unclear despite all the efforts that have been made for decades now. Nevertheless, a lot of fascinating discoveries have been made in the last few years and, despite how challenging it might be, this is for sure a very promising field of research. As more and more complete bacterial genome sequences become available and new genetic manipulation tools and other innovative techniques keep emerging, the search for new insights into the microbial physiology and ecology gets more accessible. Even though there is a large number of bacterial genes with no function prediction yet, and many homology-based predicted genes are sometimes misannotated, it is often necessary to experimentally validate their function in order to better understand the complex microbial functional systems. Additionally, some of these misannotation reports might be related to enzymatic substrate promiscuity in some cases, rather than actual specificity identification errors, as these enzymes seem to be able to catalyze different reactions. Furthermore, detection and quantification of serotonin and melatonin is still very challenging as these compounds are usually produced in low quantities and the samples are often difficult to analyze. Thus, more effective techniques (or optimization of those in use) that allow the simple and unambiguous quantification of serotonin and melatonin (and their biosynthetic intermediates) in bacteria are essential (Danilovich et al., 2021).

In conclusion, future efforts must focus on genomic analysis to provide a better understanding of the wild-type microbial genetics and possibly identify the key enzymes involved in serotonin and melatonin biosynthesis, as well as their specificity and the optimal conditions under which they are active. Considering the human gut microbiota as a possible source of serotonin and melatonin, further investigation on these indolamines’ biosynthesis in bacteria might uncover key information on these metabolic pathways. It then becomes necessary to replicate previous discoveries and translate these into human conditions, as these new findings can potentially come to revolutionize current therapeutic approaches to diseases related to serotonergic dysfunction and ideally open the path to the development of new bacteriotherapeutic strategies.

## Author Contributions

JM and NE contributed to the conceptualization. SG and JM contributed to the investigation. SG, DN-C, SC, NE, and JM contributed to the writing and final approval. SC and NE contributed to the funding. All authors contributed to the article and approved the submitted version.

## Funding

This work was funded by Portuguese national funds *via* FCT—Fundação para a Ciência e a Tecnologia under projects PTDC/MED-NEU/3644/2020, UIDB/04539/2020, UIDP/04539/2020, and LA/P/0058/2020 and PhD Fellowship SFRH/BD/117777/2016 (to DN-C).

## Conflict of Interest

The authors declare that the research was conducted in the absence of any commercial or financial relationships that could be construed as a potential conflict of interest.

## Publisher’s Note

All claims expressed in this article are solely those of the authors and do not necessarily represent those of their affiliated organizations, or those of the publisher, the editors and the reviewers. Any product that may be evaluated in this article, or claim that may be made by its manufacturer, is not guaranteed or endorsed by the publisher.

## Glossary

**Table:** 

5-HT	5-Hydroxytryptamine (serotonin)
5-HTP	5-Hydroxytryptophan
5-MT	5-Methoxytryptamine
AAAD	Aromatic amino acid decarboxylase
AAAH	Aromatic amino acid hydroxylase
ADHD	Attention deficit hyperactivity disorder
ASD	Autism spectrum disorders
ASMT	*N*-Acetylserotonin-*O*-methyltransferase
BA	Biogenic amine
BBB	Blood–brain barrier
BP	Bipolar disorder
CD	Crohn’s disease
CNS	Central nervous system
COMT	Caffeic acid *O*-methyltransferase
EC	Enterochromaffin cells
ENS	Enteric nervous system
HPLC-MS	High performance liquid chromatography mass spectrometry
IBD	Inflammatory bowel disease
IBS	Irritable bowel syndrome
L-DOPA	L-Dihydroxyphenylalanine
MH_4_	Tetrahydromonapterin
mRNA	Messenger ribonucleic acid
NADPH_2_	Nicotinamide-adenine dinucleotide phosphate (reduced)
OCD	Obsessive compulsive disorder
PEA	Phenylethylamine
PheH	Phenylalanine hydroxylase
PLP	Pyridoxal 5′-phosphate
ROS	Reactive oxygen species
SCFA	Short-chain fatty acids
SNAT	Serotonin-*N*-acetyltransferase
T5H	Tryptamine 5-hydroxylase
TrpD	Tryptophan decarboxylase
TrpH	Tryptophan hydroxylase
TyrD	Tyrosine decarboxylase
TyrH	Tyrosine hydroxylase
UC	Ulcerative colitis
